# Psittacosis chlamydia pneumonia complicated with organizing pneumonia: a case report and literature review

**DOI:** 10.3389/fmed.2025.1670456

**Published:** 2025-11-17

**Authors:** Qiao Li, Xu Sun, Wei Lei, Yehan Zhu, Wenwen Du, Xinyu Jiang, Nan Su

**Affiliations:** Department of Pulmonary and Critical Care Medicine, The First Affiliated Hospital of Soochow University, Suzhou, China

**Keywords:** organizing pneumonia, secondary organizing pneumonia, Psittacosis Chlamydia Pneumonia, case report, literature review

## Abstract

**Background:**

Secondary organizing pneumonia (SOP) may develop following infections. Psittacosis, caused by *Chlamydia psittaci* (*C. psittaci*), is a zoonotic disease transmitted from birds to humans. It can present with a wide spectrum of symptoms, ranging from mild flu-like illness to life-threatening severe pneumonia. Cases of *C. psittaci* infection complicated by organizing pneumonia (OP) are rarely reported, and delayed treatment may pose a life-threatening risk.

**Methods:**

We report a case of *C. psittaci* pneumonia complicated by OP. To identify additional cases and clarify the clinical features of this condition, a literature search was conducted using the PubMed and Embase databases for the period from January 1995 to May 2025. The search included the following keywords: “psittacosis,” “*Chlamydia psittaci*,” “chlamydia,” “organizing pneumonia,” and “bronchiolitis obliterans with organizing pneumonia.”

**Results:**

A 66-year-old male with a history of poultry farming presented with fever, cough, sputum production, and hemoptysis. Empirical antimicrobial therapy with ceftizoxime was ineffective. To identify the etiology of the pulmonary lesions, bronchoscopy was performed, and *C. psittaci* infection was confirmed by metagenomic next-generation sequencing (mNGS) of bronchoalveolar lavage fluid (BALF). Although the lesions partially resolved after moxifloxacin therapy, the patient experienced recurrent episodes. Chest CT revealed migratory lesions, which are uncommon in *C. psittaci* pneumonia. Further pathological examination of the specimen confirmed the diagnosis of OP. The patient's condition improved following corticosteroid therapy. A review of the literature indicated that none of the three previously reported cases were definitively diagnosed at initial admission; all presented primarily with fever and cough. One case progressed to severe pneumonia and resulted in death.

**Conclusion:**

OP caused by *C. psittaci* pneumonia presents with non-specific symptoms and signs, making early diagnosis challenging. During treatment of *C. psittaci* pneumonia, if empirical anti-infective therapy shows no response after three days, or if imaging reveals features such as consolidation, migratory lesions, or a reverse halo sign, the possibility of concurrent OP should be considered. Pathological examination is recommended in such cases to avoid missed diagnosis and to ensure timely intervention.

## Introduction

Organizing pneumonia (OP), previously termed bronchiolitis obliterans with organizing pneumonia (BOOP) (1), In 2002, the American Thoracic Society and the European Respiratory Society adopted the term OP to replace BOOP (2). Pathologically, OP is characterized by patchy intraluminal loose connective tissue plugs within alveolar ducts, alveoli, and bronchioles. Etiologically, it is categorized into cryptogenic organizing pneumonia (COP) and Secondary organizing pneumonia (SOP) (1, 3). SOP occurs in association with various underlying conditions, including infections (bacterial, fungal, viral, parasitic, or mycobacterial), chronic inflammatory diseases, drug toxicity, radiation therapy, or inhalation of harmful substances (1, 3, 4). Several risk factors for developing OP have been reported. In patients with COVID-19 pneumonia, being aged 50 or above, having diabetes, and hypoxemia (SpO_2_ < 88%) at admission are associated with an increased risk of OP or acute fibrinous organizing pneumonia (AFOP) (5). The presence of OP/AFOP in these patients correlates with higher rates of complications such as respiratory failure, acute kidney injury, secondary infection, pneumothorax, mediastinal emphysema, and pulmonary embolism, as well as significantly elevated 90-day mortality (5). Among immunocompromised individuals, high-risk profiles for SOP include those with hematologic malignancies, corticosteroid therapy, solid tumors (e.g., colon or hepatic carcinoma), cachexia, or arterial hypertension (6).

*Chlamydia psittaci* (*C. psittaci*) is responsible for ~1.03% of community-acquired pneumonia (CAP) cases (7), The relatively high prevalence of *C. psittaci* infections in China can be attributed to a combination of factors, including geographical and climatic conditions, occupational and lifestyle exposures, advancements in diagnostic technology, and heightened susceptibility in certain populations. The primary route of transmission is direct contact with or inhalation of contaminated avian droplets. Additional risk factors include overcrowding, age ≥65 years, poor sanitation, immunocompromised status, intensive poultry farming, and close contact with pet birds (8).

Clinical symptoms typically begin abruptly with nonspecific, flu-like manifestations such as high fever, headache, chills, malaise, and myalgia (9). Beyond the respiratory system, *C. psittaci* can also affect multiple organs—including the heart, liver, spleen, joints, meninges, and central nervous system (9), leading to complications such as respiratory failure, pericarditis, endocarditis, myocarditis, hepatosplenomegaly, anemia, acute myocardial injury, heart failure, rhabdomyolysis, disseminated intravascular coagulation, and gastrointestinal bleeding (10). In severe cases, the disease may progress rapidly to life-threatening conditions including severe pneumonia, acute respiratory distress syndrome (ARDS), sepsis, respiratory failure, and multiple organ failure (10).

Laboratory diagnosis of *C. psittaci* infection can be achieved through culture-based methods and various serological assays. However, the limited specificity and sensitivity of these conventional tests may lead to false-negative results, particularly in the early acute phase of the disease. In contrast, PCR-based detection offers a more rapid, practical, and reliable alternative (9, 10). In recent years, the introduction of metagenomic next-generation sequencing (mNGS) has further improved diagnostic accuracy and reduced underdiagnosis of psittacosis (9). As a high-throughput method, mNGS allows for the simultaneous detection of a wide range of pathogens (10). With a typical turnaround time of 24–72 h, mNGS facilitates rapid diagnosis of *C. psittaci*, helps reduce unnecessary antibiotic use, and shortens the clinical course (11). Studies have shown that early pathogen identification via mNGS enables timely treatment adjustments and is associated with improved patient prognosis (10–12).

Recent data from China suggest that *C. psittaci* infection is relatively common in the country (8). When complicated by OP, diagnosis is often delayed due to non-specific clinical manifestations, which may in turn postpone appropriate treatment. This case report describes a patient with *C. psittaci* pneumonia complicated by OP, highlighting the diagnostic and therapeutic challenges involved.

## Case presentation

A 66-year-old man was admitted to the First Affiliated Hospital of Soochow University (Suzhou, China). He had a long-term history of raising ducks at home but no other significant past illnesses. The patient denied smoking, alcohol consumption, and any family history of infectious or genetic diseases.

**Figure 1:**
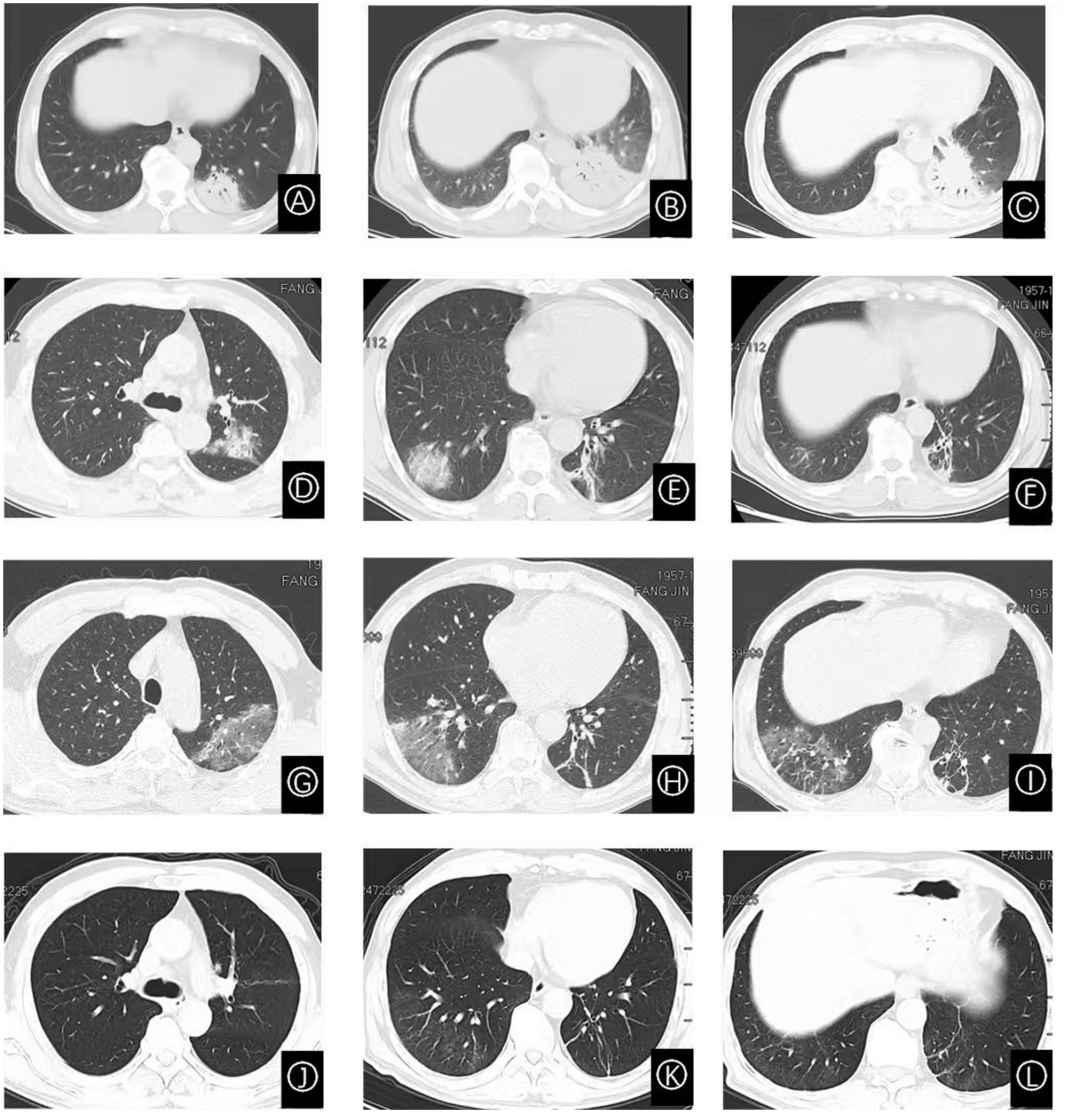
Serial chest CT images demonstrating the dynamic evolution of pulmonary lesions. **(A)** CT scan obtained on day 5 of illness shows an exudative shadow in the left lower lobe. **(B)** Scan on day 8 reveals interval enlargement of the lesion in the left lower lobe. **(C)** By day 14, the exudative shadow shows signs of absorption compared to **(B)**. **(D–F)** CT images on day 26 demonstrate new, multifocal bilateral pulmonary involvement, featuring exudative shadows, ground-glass opacities, consolidations, and migratory lesions. **(G–I)** Follow-up on day 37 shows persistent pulmonary lesions with no significant improvement. **(J–L)** Scan on day 48, following glucocorticoid therapy, shows near-complete resolution of the pulmonary abnormalities.

Nine days prior to admission, he developed a fever of unknown origin, accompanied by chills, rigors, headache, and dizziness during febrile episodes. On the fifth day of illness, a chest CT scan showed patchy dense opacities in the left lower lobe basal segment, featuring mixed ground-glass and consolidation shadows (Figure 1A). Treatment with intravenous ceftizoxime (2 g every 12 h) was initiated. However, his fever persisted and was followed by the onset of cough, sputum production, and blood-streaked sputum. A repeat chest CT revealed progression of consolidation in the same lung region (Figure 1B), prompting hospital admission.

On physical examination at admission, his body temperature was 38.5 °C (101.3°F), pulse 102 beats/min, respiratory rate 18 breaths/min, and blood pressure 139/61 mm Hg. Auscultation of the lungs demonstrated clear breath sounds bilaterally, with no audible rales or rhonchi. Initial laboratory tests included routine blood work and sputum samples for microbiological studies; detailed results are provided in Supplementary Table 1.

Upon admission, the patient presented with a temperature of 38.5 °C, pulse rate of 102 beats/min, respiratory rate of 18 breaths/min, and blood pressure of 139/61 mm Hg. Auscultation revealed clear breath sounds in both lungs without adventitious sounds. Initial laboratory investigations included routine blood tests and sputum cultures; detailed results are provided in Table 1.

**Table 1:** Major test results at the time of the patient's admission.

**Laboratory analysis**	**Level of the first admission**	**Level of the second admission**	**Normal range**
CRP	192.43	12.76	0–4 mg/L
WBC	6.34	7.47	3.50–9.50E+9/L
RBC	5.11	4.96	4.30–5.80E+12/L
HGB	153	147	130–175g/L
PLT	360	251	125–350E+9/L
NEU%	57.2	84.5	40.0–75.0%
LYM%	28.1	11.2	20.0–50.0%
CHOL	5.85	6.07	2.6–5.2 mmol/L
TG	2.71	0.86	0.34–1.70 mmol/L
HDL-C	0.62	1.09	>1.04 mmol/L
LDL-C	4.18	4.2	< =3.37 mmol/L
ALT	167	119	< =41 U/L
AST	55	29	< =40 U/L
ALB	36.2	35.5	40–55 g/L
A/G	1	1	1.2–2.4
Glu	5.66	7.8	3.9–6.1 mmol/L
UREA	5.59	6.42	3.6–9.5 mmol/L
CREA	66	57	57–111 ummol/L
UA	207	210	208–428 ummol/L
K	4.59	4.79	3.5–5.3 mmol/L
Na	142	136	137–147 mmol/L
Ca	2.21	2.26	2.11–2.52 mmol/L
Sputum Culture	Negative	–	Negative
Blood Culture	Negative	–	Negative

Due to the lack of clinical and radiographic improvement after 5 days of prior treatment, bronchoscopy was indicated and performed on the second hospital day. The procedure revealed mild narrowing of the orifices of the left lower lobe posterior basal segment and the medial subsegment of the right upper lobe. Transbronchial lung biopsy (TBLB) was performed under ultrasound guidance in the left lower lobe posterior basal segment. Pathological examination showed inflammatory cell infiltration. The mNGS of BALF detected *C. psittaci* with 5 sequence reads. BALF galactomannan testing, BALF culture, and sputum culture were all negative. Intravenous moxifloxacin (0.4 g every 24 h) was initiated. A follow-up chest CT on day 7 showed improvement in pulmonary exudates (Figure 1C). The patient was discharged with a one-week course of oral moxifloxacin.

During outpatient follow-up 9 days after discharge (illness day 26), repeat chest CT demonstrated partial resolution of left lower lobe inflammation but revealed new infiltrates in the left upper and right lower lobes (Figures 1D–F). Thirteen days post-discharge, the patient experienced recurrent fever (peak 39 °C) accompanied by rhinorrhea, though without cough or sputum. He was readmitted for further evaluation; laboratory results are shown in Table 1.

**Figure 2:**
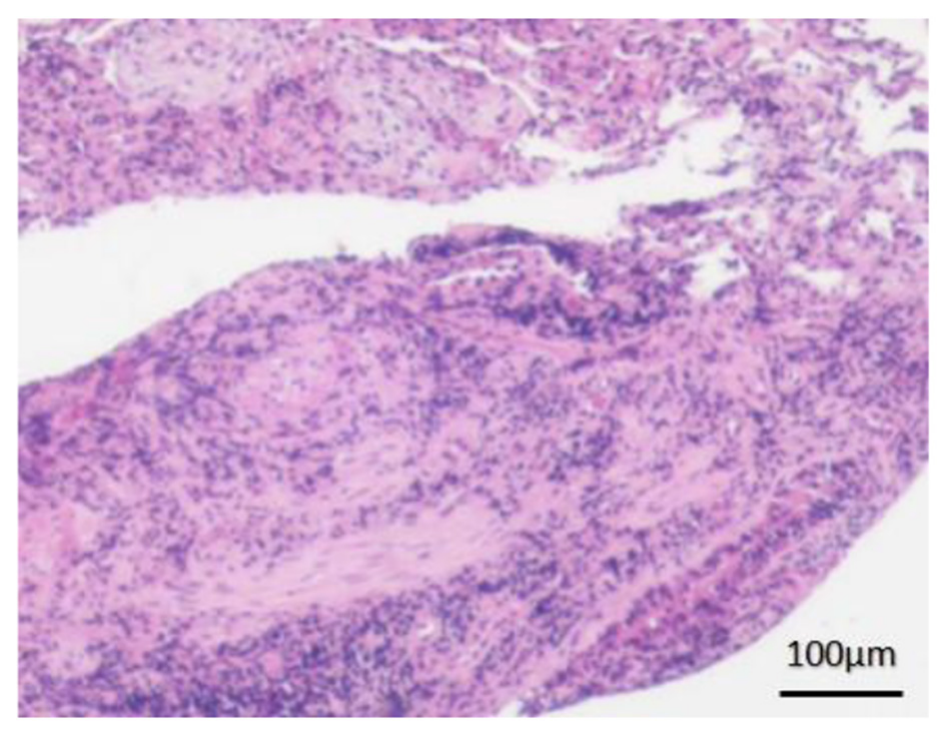
Transbronchial lung biopsy (hematoxylin and eosin staining; original magnification, 10×) showed loose, collagen-like connective tissue hyperplasia in the alveoli and alveolar ducts, with evident formation of Masson bodies.

After 11 days of minocycline (100 mg orally every 12 h), repeat CT showed no significant radiographic improvement (Figures 1G–I). Given the clinical picture of recurrent fever with migratory pulmonary lesions, the prior biopsy specimen was re-examined and pathologically diagnosed as OP (Figure 2). Methylprednisolone 40 mg daily was initiated. Doxycycline was continued throughout this period due to the concomitant *C. psittaci* infection. After 10 days of combined therapy (illness day 48), chest CT showed near-complete resolution of bilateral pulmonary infiltrates (Figures 1J–L). Corticosteroid tapering was initiated 2 weeks later at a rate of 5 mg per week until discontinuation.

The patient was advised to avoid re-exposure to potential infection sources such as ducks, chickens, parrots, and pigeons. Recommendations included regular quarantine of domestic birds, use of personal protective equipment when contact is unavoidable, maintaining good indoor ventilation, consistent hand hygiene, and respiratory etiquette. At the one-year outpatient follow-up, the patient remained asymptomatic and reported no psychological sequelae from the illness.

## Literature review

### Search strategy

To identify other cases of Psittacosis Chlamydia Pneumonia Complicated with Organizing Pneumonia, and to clarify the clinical features of this disease, we searched the PubMed, Embase, and Web of Science databases using the keywords (“psittacosis” OR “*Chlamydia psittaci*” OR “chlamydia”) AND (“organizing pneumonia” OR “bronchiolitis obliterans with organizing pneumonia”).

### Inclusion and exclusion criteria

Studies included were English-language reports of Psittacosis Chlamydia Pneumonia Complicated with BOOP or OP.

### Data extraction and analysis

To ensure comprehensive and non-redundant data collection, all identified articles were meticulously reviewed. Relevant information was extracted and compiled as descriptive statistics in an Excel spreadsheet.

## Results

The initial literature search yielded five articles. After excluding two non-English publications, three studies met the inclusion criteria, collectively reporting three cases of *C. psittaci* infection complicated by OP.

As summarized in Table 2, all three reported patients were male and presented with symptoms including fever, cough, myalgia, sore throat, dyspnea, and lethargy. Initial microbiological cultures were negative in all cases. The diagnosis was ultimately confirmed by bronchoscopic lung biopsy, supported by serological and/or molecular testing. Notably, none of the patients were diagnosed during their initial medical presentation; a definitive diagnosis was achieved only after pathological examination following unsuccessful empirical antibiotic therapy.

**Table 2:** Case reports of Psittacosis Chlamydia Pneumonia complicated with organizing pneumonia.

**Country**	**Sex/Age**	**Past health**	**Contact with poultry**	**Symptoms**	**Physicalexamination**	**Respiratory failure**	**Markers ofinflammation**	**Imaging**	**Bronchoscopyexamination**	**Pathogen detection**	**First visit for diagnosis/Diagnostic time**	**Glucocorticoid**	**Antibiotics after pathogen identification**	**Result**
France (13)	M/70	Prostate adenoma	N/A	Fever, cough, severe sore hroat, worsening dyspnea	Sparse crackles	Yes	WBC 7.8 cells × 10^9^/L, with 71% NEU	Diffuse alveolar infiltrates in the right and middle lobes, thickening of the bronchial wall, and a small pleural effusion on the right side	Granulation tissue in the distal airways	Chlamydia group complement-fixation test and immunofluorescence test	NO/Day 53 of the illness course	Prednisone 1mg·kg-1·day-1, treatment course more than 1 year	Doxycycline for 21 days, and then, Erythromycin was added to doxycycline	Improvement
Australia (14)	M/63	Chronic urinary tract infections and anxiety	N/A	Fevers, myalgia, lethargy, anorexia, dry cough	Decreased breath sounds	Yes	CRP 456 mg/dL, WBC 12.4 × 10^9^/L	Consolidation of the left lung with moderate left-sided pleural effusion	Bronchoalveolar lavage fluid with a small amount of neutrophils and macrophages	PCR	NO/Day 33 of the illness course	Prednisone 50mg	doxycycline for 23 days, and then, Meropenem and vancomycin were added, piperacillin-tazobactam	Death
China (15)	M/56	N/A	No	fever, chills	No rales in both lungs	No	WBC 7.39 × 10^9^/L, 80% NEU, CRP 127.74 mg/L, ESR 101 mm/h	The left lower lobe has a plaque-like, high-density opacity with an air bronchogram	Chronic inflammation of the respiratory mucosa, submucosal fibrovascular hyperplasia, and tissue pneumonia changes in the surrounding lung tissue	BALF mNGS	NO/Day 28 of the illness course	Methylprednisolone 40mg, reduce the dosage by 4mg every two weeks until discontinuation	Moxifloxacin for about 1 months	Improvement

M, male; PCR, polymerase chain reaction; CRP, C-reactive protein; ESR, erythrocyte sedimentation rate; BALF, bronchoalveolar lavage fluid; mNGS, metagenomic next-generation sequencing.

All patients received a combination of antibiotic and glucocorticoid therapy. Although two cases progressed to respiratory failure, clinical improvement was observed in two patients following this combined regimen. One patient, however, died due to a concurrent *Pseudomonas aeruginosa* infection (13–15).

## Discussion

Psittacosis is a systemic zoonotic illness caused by *C. psittaci*, primarily transmitted to humans through inhalation of infectious aerosols from birds, with avian contact being the principal risk factor (9, 16). The present case involved a patient with a history of duck farming who developed infection consistent with this exposure. Following admission, the failure of prior beta-lactam antibiotic therapy prompted further investigation. Bronchoscopy with BALF mNGS successfully identified *C. psittaci* as the causative pathogen. When managing patients with respiratory infections, if conventional diagnostic methods fail to identify a pathogen within 3 days, empirical antimicrobial therapy proves ineffective, or imaging shows deterioration or new lesions, we recommend obtaining respiratory specimens for mNGS testing. This approach not only aids in detecting atypical pathogens but can also provide information on the presence of antimicrobial resistance genes (17).

In this case, however, the patient's condition recurred after initial antimicrobial treatment. Imaging demonstrated migratory pulmonary infiltrates, and subsequent pathological examination confirmed OP. Clinical improvement was achieved only after initiating glucocorticoid therapy. Similar to the three other cases identified in our literature review, the non-specific clinical features of OP associated with *C. psittaci* infection often lead to diagnostic delays. Such delays can complicate management and increase mortality risk. Among the reviewed cases, two patients developed respiratory failure; one of them presented with pneumothorax, possibly secondary to a bronchopleural fistula, and ultimately died.

*C. psittaci* infection commonly presents with fever, severe headache, myalgia, fatigue, dyspnea, chills, and dry cough (9, 16). Some patients may also experience abdominal pain, diarrhea, hemoptysis, vomiting, anorexia, sore throat, nasal congestion, rhinorrhea, convulsions, or coma (18). OP often manifests with non-specific symptoms such as dry cough, flu-like illness, and exertional dyspnea. Additional signs may include fever, fatigue, weight loss, hemoptysis, arthralgia, and night sweats. Physical examination may reveal crackles, bronchial breath sounds, and hypoxemia (1, 3). Due to the overlapping and non-specific nature of symptoms between OP and infectious processes, diagnosis is frequently delayed. Based on the present case and literature review, fever and cough appear to be the most common symptoms in cases of *C. psittaci*-associated OP (13–15).

The severity of *C. psittaci* pneumonia is highly variable. Extrapulmonary involvement may affect the cardiovascular, digestive, and musculoskeletal systems (9), leading to complications such as respiratory failure, heart failure, myocardial injury, hepatosplenomegaly, anemia, rhabdomyolysis, disseminated intravascular coagulation, and gastrointestinal bleeding (10). Less commonly reported manifestations include skin purpura, cyanosis (19), and meningitis (20). In pregnant women, severe infection may result in stillbirth (21). There are also rare reports of acute gastroenteritis following ingestion of undercooked pigeon meat or offal (22). In severe cases, rapid clinical deterioration due to respiratory failure or multi-organ dysfunction can be fatal (10). To date, reported cases of *C. psittaci* pneumonia complicated by OP remain rare, with one documented fatality (14). These findings underscore the critical importance of early diagnosis and timely intervention in *C. psittaci* pneumonia.

Furthermore, there are currently no specific laboratory markers for OP. Approximately half of all cases present with leukocytosis, and systemic inflammatory markers—such as C-reactive protein (CRP) and erythrocyte sedimentation rate (ESR)—are commonly elevated. Pulmonary function tests often show mild to moderate restrictive ventilatory defects with impaired diffusion capacity (3). In cases of *C. psittaci* infection, patients may also exhibit elevated white blood cell and neutrophil counts, along with increased CRP and ESR. Some individuals show elevated liver enzymes (AST, ALT) and cardiac enzymes (creatine kinase), as well as reduced lymphocyte counts (23). The absence of specific laboratory indicators for both OP and *C. psittaci* infection complicates early diagnosis during the initial admission.

Radiologically, approximately three-quarters of OP patients present with multifocal consolidations, typically appearing as patchy opacities in the peripheral lower lung zones or along bronchovascular bundles. These may be associated with air bronchograms, scattered ground-glass opacities, or small nodules. A minority of cases present predominantly or exclusively with ground-glass opacities and a cobblestone appearance (3). In psittacosis pneumonia, the most common radiographic patterns are lobar or round-type pneumonias, often accompanied by interstitial changes (24, 25). Frequent findings include intralobular lines, air bronchogram signs, and reversed halo signs (24). Multilobar consolidation or ground-glass infiltrates are also common, and some patients may develop pleural or pericardial effusions (18).

Pathology remains the gold standard for diagnosing OP. Histopathologically, OP is characterized by the presence of fibroblastic and myofibroblastic plugs within alveolar spaces and distal bronchioles, often intermixed with inflammatory cells such as lymphocytes, plasma cells, and macrophages (3). Bronchoscopy with BALF is a valuable diagnostic procedure. BALF typically shows a mixed cellular pattern with elevated lymphocytes, neutrophils, and/or eosinophils, and helps exclude infection, malignancy, or other inflammatory conditions (1, 3). A definitive diagnosis should integrate clinical presentation, imaging features, BALF findings, and pathological results, while systematically ruling out alternative diagnoses. In cases where bronchoscopic biopsies yield insufficient tissue, a surgical lung biopsy may be necessary to establish the diagnosis.

During the treatment of *C. psittaci* pneumonia, if imaging studies fail to rule out concurrent OP or the diagnosis remains unclear, bronchoscopy, bronchoalveolar lavage fluid analysis, and transbronchial biopsy can aid in diagnosis and help differentiate from other diseases. However, pathological differentiation between COP and SOP remains challenging (3). The diagnosis of SOP requires integration of clinical history—such as recent respiratory infections, underlying systemic diseases, or known treatments associated with OP development—with chest imaging findings, significantly enhancing the likelihood of an accurate diagnosis. Regardless, continuous longitudinal follow-up of clinical and chest imaging characteristics remains essential.

The management of psittacosis requires targeted antimicrobial therapy guided by timely pathological and microbiological confirmation. Tetracyclines, macrolides, and fluoroquinolones have demonstrated efficacy against *C. psittaci*, with a typical treatment duration of 10–14 days (16). Tetracyclines are considered the first-line therapeutic option (16). For OP, glucocorticoids constitute the standard treatment. Although no universally established dosing regimen exists, prednisone is commonly initiated at 0.5–1.5 mg/kg per day, followed by a gradual taper—typically reduced by 10 mg every 4 weeks—and eventually discontinued over a total course of 6–12 months (26). In cases of secondary OP (SOP) complicating *C. psittaci* pneumonia, the optimal glucocorticoid dosage and treatment duration remain unclear. In the present case, methylprednisolone was administered at a dose equivalent to 1 mg/kg/day of prednisone, consistent with the regimen reported by Diehl et al. (13). While many studies do not detail glucocorticoid-related adverse events in OP treatment, some reports indicate adverse reaction rates as high as 53% (27). Commonly observed side effects include weight gain, myopathy, osteoporosis, hypertension, and systemic infections (28).

In summary, for patients who respond poorly to empirical antimicrobial therapy, mNGS may be considered to facilitate pathogen identification. Early diagnosis of SOP following *C. psittaci* pneumonia remains challenging. If patients show inadequate clinical improvement after appropriate antibiotic therapy or exhibit migratory pulmonary lesions on imaging, the possibility of concurrent OP should be actively considered to avoid missed diagnosis. Specifically, when empirical anti-infective treatment fails after 3 days, or when chest imaging demonstrates features such as consolidation, migratory opacities, or a reverse halo sign, the potential coexistence of OP should be evaluated. In cases where pathological confirmation is difficult to obtain due to disease severity or technical limitations, the judicious use of corticosteroids may be considered, provided that close monitoring for potential adverse effects is implemented.

## Data Availability

The original contributions presented in the study are included in the article/supplementary material, further inquiries can be directed to the corresponding author.

## References

[1] Ketchersid K. A review of organizing pneumonia. JAAPA. (2023) 3:16–9. doi: 10.1097/01.JAA.0000918776.59717.eb 36749158

[2] American Thoracic Society; European Respiratory Society. American Thoracic Society/European Respiratory Society International Multidisciplinary Consensus Classification of the Idiopathic Interstitial Pneumonias. This joint statement of the American Thoracic Society (ATS), and the European Respiratory Society (ERS) was adopted by the ATS board of directors, June 2001 and by the ERS Executive Committee, June 2001. Am J Respir Crit Care Med. (2002) 165:277–304. doi: 10.1164/ajrccm.165.2.ats01 11790668

[3] Cherian SV Patel D Machnicki S Naidich D Stover D Travis WD . Algorithmic approach to the diagnosis of organizing pneumonia: a correlation of clinical, radiologic, and pathologic features. Chest. (2022) 162:156–78. doi: 10.1016/j.chest.2021.12.659 35038455 PMC9899643

[4] Limkul L Tovichien P. Secondary organizing pneumonia after infection. World J Clin Cases. (2024) 12:6877–82. doi: 10.12998/wjcc.v12.i36.6877 39726927 PMC11531977

[5] Aikwanich A Eksombatchai D Petnak T Tassaneeyasin T Boonsarngsuk V. Risk factors for secondary organizing pneumonia and acute fibrinous and organizing pneumonia in patients with COVID-19 pneumonia. Infect Drug Resist. (2024) 17:5017–26. doi: 10.2147/IDR.S481540 39554470 PMC11566205

[6] Terrabuio Junior AA Parra ER Farhat C Capelozzi VL. Autopsy-proven causes of death in lungs of patients immunocompromised by secondary interstitial pneumonia. Clinics. (2007) 62:69–76. doi: 10.1590/S1807-59322007000100011 17334552

[7] Hogerwerf L De Gier B Baan B Van Der Hoek W. Chlamydia psittaci (psittacosis) as a cause of community-acquired pneumonia: a systematic review and meta-analysis. Epidemiol Infect. (2017) 145:3096–105. doi: 10.1017/S0950268817002060 28946931 PMC9148753

[8] Ghasemian A Pezeshki B Memariani M Mahmoodi S Kohansal M Rajabi-Vardanjani H. The emergence potential of Chlamydia psittaci and chlamydia felis as zoonotic agents causing ocular and respiratory infections in humans and animals. Arch Razi Inst. (2024) 79:685–94. doi: 10.32592/ARI.2024.79.4.685 40256594 PMC12004038

[9] Wang J Wang B Xiao J Chen Y Wang C. Chlamydia psittaci: a zoonotic pathogen causing avian chlamydiosis and psittacosis. Virulence. (2024) 15:2428411. doi: 10.1080/21505594.2024.2428411 39541409 PMC11622591

[10] Liu K Wu L Chen G Zeng D Zhong Q Luo L . Clinical characteristics of Chlamydia psittaci infection diagnosed by metagenomic next-generation sequencing: a retrospective multi-center study in Fujian, China. Infect Drug Resist. (2024) 17:697–708. doi: 10.2147/IDR.S443953 38405056 PMC10894596

[11] Huang M Wang Y Lu Y Qu W Zou Q Zhang D . Clinical cracteristics and predicting disease severity in *Chlamydia psittaci* infection based on metagehanomic next-generation sequencing. Infect Drug Resist. (2025) 18:1171–81. doi: 10.2147/IDR.S509879 40027914 PMC11872090

[12] Xiao Q Shen W Zou Y Dong S Tan Y Zhang X . Sixteen cases of severe pneumonia caused by *Chlamydia psittaci* in South China investigated via metagenomic next-generation sequencing. J Med Microbiol. (2021) 70:10. doi: 10.1099/jmm.0.001456 34817316

[13] Diehl JL Gisselbrecht M Meyer G Israel-Biet D Sors H. Bronchiolitis obliterans organizing pneumonia associated with chlamydial infection. Eur Respir J. (1996) 9:1320–2. doi: 10.1183/09031936.96.09061320 8804955

[14] Zuzek R Green M May S. Severe psittacosis progressing to suspected organizing pneumonia and the role of corticosteroids. Respir Med Case Rep. (2021) 34:101486. doi: 10.1016/j.rmcr.2021.101486 34381682 PMC8339220

[15] Gao Y Zhang X Liu J Gong L Chen G Zhou X. Chlamydia psittaci pneumonia complicated with organizing pneumonia: a case report and literature review. IDCases. (2022) 30:e01584. doi: 10.1016/j.idcr.2022.e01584 36193103 PMC9526182

[16] Stewardson AJ Grayson ML. Psittacosis. Infect Dis Clin North Am. (2010) 24:7–25. doi: 10.1016/j.idc.2009.10.003 20171542

[17] CMLC Association Society I.D.G.L. Chinese expert consensus on diagnosis and treatment of psittacosis. Chin J Clin Infect Dis. (2024) 17:191–204. doi: 10.3760/cma.j.issn.1674-2397.2024.03.002

[18] Li X Xiao T Hu P Yan K Wu J Tu X . Clinical, radiological and pathological characteristics of moderate to fulminant psittacosis pneumonia. J PLoS ONE. (2022) 17:e0270896. doi: 10.1371/journal.pone.0270896 35816485 PMC9273088

[19] Meijer R van Biezen P Prins G Boiten HJ. Multi-organ failure with necrotic skin lesions due to infection with *Chlamydia psittaci*. *Int J Infect Dis*. (2021) 106:262–4. doi: 10.1016/j.ijid.2021.03.091 33823280

[20] Shi Y Chen J Shi X Hu J Li H Li X . A case of *chlamydia psittaci* caused severe pneumonia and meningitis diagnosed by metagenome next-generation sequencing and clinical analysis: a case report and literature review. BMC Infect Dis. (2021) 21:621. doi: 10.1186/s12879-021-06205-5 34193063 PMC8243071

[21] Paul L Comstock J Edes K Schlaberg R. Gestational Psittacosis resulting in neonatal death identified by next-generation RNA sequencing of postmortem, formalin-fixed lung tissue. Open Forum Infect Dis. (2018) 5:ofy172. doi: 10.1093/ofid/ofy172 30151406 PMC6105100

[22] Deng H Liang X Dang S Qiu J. *Chlamydia psittaci* enteric infection in an adult man: a case report. Int J Infect Dis. (2025) 158:107967. doi: 10.1016/j.ijid.2025.107967 40571117

[23] Li Y Lin F Li W Chen G Li S Liu B . Comparison of clinical, laboratory and radiological characteristics between *Chlamydia psittaci* and adenovirus pneumonias: a multicenter retrospective study. Int J Infect Dis. (2023) 126:114–24. doi: 10.1016/j.ijid.2022.11.029 36455811

[24] Yang N Ou Z Sun Q Pan J Wu J Xue C. *Chlamydia psittaci* pneumonia - evolutionary aspects on chest CT. BMC Infect Dis. (2025) 25:11. doi: 10.1186/s12879-024-10374-4 39748281 PMC11697637

[25] Zhang S Fu Y Fang L Xu Q Gu S Zhou H . Psittacosis pneumonia with the reversed halo sign: a case report and literature review. BMC Infect Dis. (2025) 25:717. doi: 10.1186/s12879-025-11081-4 40382544 PMC12085835

[26] Drakopanagiotakis F Polychronopoulos V Judson MA. Organizing pneumonia. Am J Med Sci. (2008) 335:34–9. doi: 10.1097/MAJ.0b013e31815d829d 18195581

[27] Barroso E Hernandez L Gil J Garcia R Aranda I Romero S. Idiopathic organizing pneumonia: a relapsing disease. 19 years of experience in a hospital setting. Respiration. (2007) 74:624–31. doi: 10.1159/000103240 17536184

[28] Cendon L Rafecas Codern A de la Rosa D Castellví I Spagnolo P Castillo D. Systematic review of systemic corticosteroids for treatment of organizing pneumonia. Open Respir Arch. (2022) 4:100211. doi: 10.1016/j.opresp.2022.100211 37496960 PMC10369534

