# Supplementary figures and images for: Psittacosis chlamydia pneumonia complicated with organizing pneumonia: a case report and literature review
Source: Front Med (Lausanne). 2025 Nov 17;12:1670456. doi: 10.3389/fmed.2025.1670456 (PMC12665588; doi:10.3389/fmed.2025.1670456)

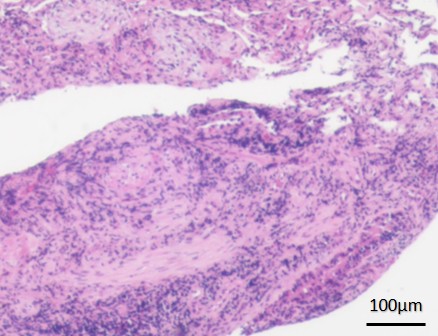

Supplement: Supplementary file 2 [file Image_1.jpeg]
